# Differential Responses in *Wedelia trilobata* and *Wedelia chinensis* Under Light Stress: Roles of Abscisic Acid and Sodium Tungstate

**DOI:** 10.3390/plants15101570

**Published:** 2026-05-21

**Authors:** Ping Huang, Bin Li, Lili He, Yijie Chen, Hong Yang, Adeel Abbas

**Affiliations:** 1 Institute of Environment and Ecology, School of Environment and Safety Engineering, Jiangsu University, Zhenjiang 212013, China; 2 School of Emergency Management, Jiangsu University, Zhenjiang 212013, China

**Keywords:** *Wedelia trilobata*, invasive plants, light intensity, abscisic acid (ABA), physiological plasticity

## Abstract

Light availability is a primary determinant of plant growth and a key factor influencing the success of alien plant invasions. Although the phytohormone abscisic acid (ABA) is a known master regulator of abiotic stress responses, its specific role in the shade tolerance and competitive advantage of invasive species remains poorly understood. In this study, we conducted a comparative experiment using the highly invasive *Wedelia trilobata* and its native congener, *Wedelia chinensis*. We investigated their eco-physiological responses to normal (100%) and low-light (30%) intensities, coupled with the application of exogenous ABA (A1) and the biosynthesis inhibitor sodium tungstate (S1). Our results showed that low light significantly inhibited the growth and photosynthetic capacity of both species, reducing biomass and net photosynthetic rate *P_n_*. However, exogenous ABA application specifically enhanced the *P_n_* and biomass of the invasive *W. trilobata* under low-light stress, while reducing malondialdehyde (MDA) content and optimizing antioxidant enzyme activities (SOD, POD, and CAT). Conversely, the inhibition of endogenous ABA by sodium tungstate exacerbated oxidative damage and photosynthetic decline in both species, with a more pronounced negative impact on *W. trilobata*. Correlation analysis further revealed that *W. trilobata* maintains a superior capacity to coordinate stomatal regulation and antioxidant defense through ABA signaling. These findings suggest that the invasive success of *W. trilobata* in fluctuating light environments is significantly driven by its high sensitivity and efficiency in ABA-mediated physiological plasticity, providing a potential target for managing its spread through hormonal or metabolic interference.

## 1. Introduction

Alien plant invasion has emerged as a critical driver of global biodiversity loss and ecosystem degradation, causing severe ecological and economic damage worldwide [1,2]. The remarkable ecological success of invasive plants is largely attributed to their profound phenotypic plasticity and rapid adaptation to fluctuating abiotic stresses, such as extremes in temperature, moisture, and light availability [3,4]. Among these environmental filters, light intensity acts as a primary determinant of plant growth, resource allocation, and interspecific competition [5]. In natural habitats, light conditions are highly dynamic: shading from forest canopies, riparian vegetation, or water turbidity can severely limit photosynthetic capacity and induce physiological stress [6,7]. Emerging ecological evidence indicates that highly invasive species often possess superior morphological and physiological adjustments to suboptimal light conditions compared to their native counterparts [8]. This robust shade tolerance or light-adaptive plasticity allows invaders to colonize a broader range of ecological niches, facilitating their rapid spread across diverse and often disturbed habitats [9].

To cope with such environmental constraints, plants have evolved sophisticated biochemical defense mechanisms, with the phytohormone abscisic acid (ABA) serving as a master regulatory hub [10]. While classically recognized for mediating stomatal closure during drought, modern research has firmly established ABA as a comprehensive coordinator of systemic responses to various abiotic stressors. Biochemically, ABA plays an indispensable role in mitigating stress-induced oxidative damage [11]. Exogenous application of ABA has been shown to significantly enhance the activities of key antioxidant enzymes—including superoxide dismutase (SOD), peroxidase (POD), and catalase (CAT)—thereby scavenging excessive reactive oxygen species and preserving Photosystem II efficiency under severe stress [12,13]. Crucially, this robust ABA-mediated regulatory capacity is increasingly recognized as a driving physiological factor in the success of invasive plant species. Invasive plants frequently exhibit heightened sensitivity in their ABA signaling pathways, enabling them to mount faster and more efficient stress-adaptive responses than their non-invasive relatives [14,15].

Furthermore, recent transcriptomic and physiological studies highlight an intricate crosstalk between light signaling and ABA regulatory networks. Changes in light intensity and quality can directly modulate ABA biosynthesis and signal transduction, suggesting that ABA acts as a critical integrator of light cues and physiological stress responses [16,17]. For instance, phytochrome B (phyB) interacts with ABA signaling components to balance growth and stress tolerance under variable light conditions [18]. Additionally, low light can suppress the expression of ABA biosynthesis genes such as *NCED3*, thereby reducing endogenous ABA levels and delaying stress acclimation [19]. The circadian clock also tightly gates ABA signaling, ensuring that stress responses are prioritized during specific photoperiods [20]. These findings collectively support the view that ABA acts as an integrator of light cues and physiological stress responses. To rigorously elucidate the protective mechanisms of endogenous ABA under these fluctuating environments, pharmacological inhibitors of ABA biosynthesis, such as sodium tungstate, serve as indispensable experimental tools. Sodium tungstate effectively suppresses stress-induced ABA accumulation by specifically blocking ABA-aldehyde oxidase activity, the final step in the ABA biosynthesis pathway [21]. By artificially depleting endogenous ABA levels during stress events, researchers have consistently observed exacerbated photosynthetic decline, severe oxidative damage, and compromised morphological adaptations in various plant species [22,23]. This targeted inhibition directly underscores that rapid endogenous ABA synthesis is essential for initiating effective physiological survival strategies.

Despite these molecular and physiological insights, the specific interactive effects of light intensity and ABA regulation on the competitive advantage of invasive plants remain poorly understood. *Wedelia trilobata*, a perennial clonal herb native to tropical America, has aggressively invaded diverse environments globally, particularly in regions like South China. Driven by its rapid vegetative propagation and robust physiological adaptability, *W. trilobata* severely suppresses native flora and disrupts regional biodiversity, serving as an excellent model for invasion biology [24,25]. In the present study, we utilized the highly invasive *W. trilobata* alongside its native congener, *Wedelia chinensis*, as a comparative system. Building upon our previous findings regarding ABA-mediated regulation under salt stress [26], the present study represents a comprehensive extension aimed at deciphering the role of ABA in light-induced physiological plasticity. While salt stress primarily triggers osmotic and ionic responses, light intensity fluctuations necessitate distinct acclimation strategies for carbon gain. To bridge this knowledge gap, we investigated the impacts of low-light stress from two interconnected perspectives: exogenous hormonal application and endogenous regulatory profiling. This dual approach allows us to dissect whether and how ABA signaling confers a competitive advantage to *W. trilobata* under shaded conditions—a dimension that transcends the scope of our prior investigations into salinity. By systematically applying exogenous ABA and the biosynthesis inhibitor sodium tungstate under normal and low-light intensities, we aim to address two primary research questions: (1) How do the eco-physiological responses—specifically growth performance and photosynthetic capacity—of these invasive and native species differ under light stress when ABA pathways are externally modulated? (2) Does the invasive *W. trilobata* maintain a competitive advantage through a superior capacity to regulate its antioxidant defense system during this process?

## 2. Materials and Methods

### 2.1. Plant Materials and Growth Conditions

Stem segments of the invasive *W. trilobata* were collected from Nanning, Guangxi Zhuang Autonomous Region, China (22°48′ N, 108°22′ E), and the native *W. chinensis* were obtained from a greenhouse at Jiangsu University (32°12.02′ N, 119°31.76′ E). Healthy and uniform regenerated stem segments, each retaining two nodes, were selected for the experiment. The segments were washed five times with sterile water and then soaked in a 0.1-strength Hoagland–Arnon nutrient solution for 3–4 days until adventitious roots emerged to ensure normal growth.

### 2.2. Experimental Design and Treatments

The experiment was conducted in a controlled plant growth room (greenhouse) at Jiangsu University. The environmental conditions were maintained at a relative humidity (RH) of 60%, a temperature of 25 °C, and a photoperiod of 16 h/8 h (light/dark). Rooted cuttings were planted vertically into round plastic pots (90 × 60 × 80 mm; two plants per pot). Each pot contained 350 g of a sterilized, washed, and dried mixture of river sand and vermiculite (5:1, *v*/*v*). Plants were watered thoroughly and allowed to acclimatize. Treatment commenced 4–5 days later, once young shoots had emerged. Abscisic acid (Cat. No. A4906) and sodium tungstate dihydrate (Cat. No. 223336) used in this study were purchased from Sigma-Aldrich (St. Louis, MO, USA).

A pot experiment was conducted using a full-factorial design with six treatments and 20 replicates (pots) per treatment. Pots were randomly assigned to positions within the growth chamber using a random number generator, and their positions were re-randomized every 7 days to minimize any potential positional effects. Two light intensity levels were established in the greenhouse: normal light (100% light condition, approximately 450 μmol m^−2^ s^−1^) and low light (30% light condition, approximately 135 μmol m^−2^ s^−1^). The low-light conditions were achieved using shading nets, and the light intensity was verified using a CL-200 quantum meter. Under each light condition, plants were subjected to three treatments: distilled water with the same amount of ethanol as the A1 (CK), 15 μM abscisic acid (A1), and 100 μM sodium tungstate (S1). The selected concentration of abscisic acid and sodium tungstate was based on our preliminary dose–response trials, as this level exerts a noticeable plant growth impact and endogenous hormonal profiles on both plant species. The ABA stock solution was prepared by dissolving ABA in a minimal volume of 95% ethanol and then diluted with distilled water to the working concentration of 15 μM. Sodium tungstate was directly dissolved in distilled water without additional solvent. To eliminate potential solvent interference, two control groups were initially established: a distilled water control and a solvent control (distilled water containing the same amount of ethanol as A1). Preliminary tests indicated no significant differences between these two controls across all measured parameters (*p* > 0.05). Consequently, the solvent control was used as the universal control (CK) for all subsequent treatments.

Plants in each pot received 40 mL of their respective treatment solutions every seven days, supplemented with 0.5-strength Hoagland–Arnon solution every three days to maintain optimal growth. ABA stock solutions and working solutions were prepared freshly before use and stored in amber glass bottles at 4 °C to prevent photodegradation and thermal decomposition. To further minimize light-induced degradation during the treatment process, solutions were applied to plants in the early morning (7–8 a.m.) under low-intensity light conditions. This allowed the ABA to be absorbed by the plant tissues before exposure to peak diurnal light intensity.

### 2.3. Measurement of Morphological Parameters and Biomass

After two months of growth, the plants were harvested. The aboveground and belowground parts of *W. trilobata* and *W. chinensis* were separated. Root length, plant height, and leaf area were quantified using ImageJ software (version 1.54, National Institutes of Health, Bethesda, MD, USA). The number of leaves and lateral roots was counted manually. The fresh weights of leaves, stems, and roots were recorded using an analytical balance. The samples were then dried in an oven at 65 °C for approximately 72 h to constant weight to determine the aboveground biomass, belowground biomass, and total biomass. The root-to-shoot ratio was subsequently calculated.

### 2.4. Photosynthetic Parameters and Chlorophyll Content

Net photosynthetic rate (*P_n_*), stomatal conductance (*g_s_*), transpiration rate (*T_r_*), and intercellular CO_2_ concentration (*C_i_*) were measured using a Li-6400 portable photosynthesis system (LI-COR, Lincoln, NE, USA) [27]. During gas exchange measurements, the leaf temperature in the chamber was monitored and remained relatively stable, ranging from 26.2 to 28.5 °C. To minimize the impact of diurnal temperature fluctuations, measurements were performed on fully expanded, uniform leaves from the middle section of both species between 9 and 11 a.m. on clear days in a randomized sequence across all treatment groups. Chlorophyll content (expressed as SPAD values) was measured using an FK-YL02 portable chlorophyll meter(Shandong Fangke Instrument Co., Ltd., Weifang, Shandong, China) [25]. For each plant, two symmetrical leaves from the same node were selected, and each leaf was measured twice; the average value was recorded as a single replicate for data analysis.

### 2.5. Determination of Antioxidant Enzyme Activities and MDA Content

For physiological analysis, 0.1 g of fresh leaf tissue was homogenized in 5 mL of pre-cooled 50 mM phosphate buffer (pH 7.8) containing 1% (*w*/*v*) polyvinylpolypyrrolidone (PVPP). The homogenization was performed on ice to maintain enzyme stability. The homogenate was centrifuged at 12,000× *g* for 20 min at 4 °C, and the resulting supernatant was collected and stored at 4 °C for immediate assays of enzyme activities and malondialdehyde (MDA) content. In this study, antioxidant enzyme activities were normalized by fresh weight (FW). The MDA content was determined using the thiobarbituric acid (TBA) method. For the antioxidant system, peroxidase (POD) activity was measured using the guaiacol colorimetric method; catalase (CAT) activity was determined via the ultraviolet absorption method; and superoxide dismutase (SOD) activity was assayed using the nitroblue tetrazolium (NBT) photoreduction method [26].

### 2.6. Statistical Analysis

Statistical analyses were performed using the SPSS software (version 27.0, IBM Corp., Armonk, NY, USA). The data were subjected to one-way analysis of variance (ANOVA) and two-way ANOVA to evaluate the independent and interactive effects of the treatments. Pearson’s correlation analysis was also conducted to explore relationships between parameters. All data are presented as mean ± standard error (SE) based on three biological replicates (*n* = 3). Significant differences among treatment means were determined using Duncan’s multiple range test (*p* < 0.05). All graphs were generated using Origin 2021 (version 8.1, OriginLab Corp., Northampton, MA, USA). In the figures, different lowercase letters indicate statistically significant differences at *p* < 0.05.

## 3. Results and Analysis

### 3.1. The Effects of Abscisic Acid on the Growth of Two Plant Species Under Different Light Intensities

**Figure 1:**
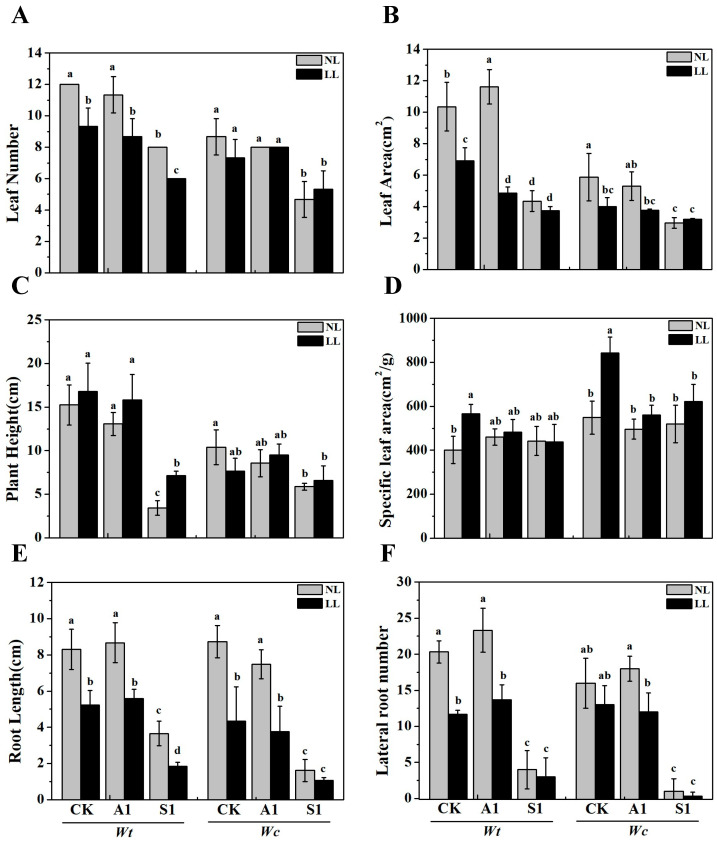
Effects of exogenous ABA and sodium tungstate dihydrate on the growth index of *W. trilobata* and *W. chinensis* under different light intensity conditions. (**A**) Leaf number; (**B**) leaf area; (**C**) plant height; (**D**) specific leaf area; (**E**) root length; and (**F**) lateral root number. NL: Normal light, LL: Low light; CK: distilled water with the same amount of ethanol as the A1, A1: 15 μM ABA, S1: 100 μM sodium tungstate dihydrate; Data are presented as mean ± SE (*n* = 3). Different lowercase letters indicate significant differences among treatments according to Duncan’s multiple range test (*p* < 0.05).

As shown in Figure 1, low-light intensity inhibited the growth of both *W. trilobata* and *W. chinensis*. The application of ABA (A1) resulted in a notable decrease in the leaf number and leaf area of both species under low-light conditions when compared to normal light, but it had no significant impact on root length or lateral root number (Figure 1A,B,E,F). The low-light treatment had a more pronounced effect on *W. trilobata*, with reductions of 31.33% in plant leaf number, 58.00% in leaf area, 55.98% in root length, and 80.32% in lateral root number compared to normal light conditions. The native species *W. chinensis* showed significant reductions in root length (31.34%) and lateral root number (67.00%) under low-light intensity (Figure 1A,B,E,F). Additionally, the S1 (sodium tungstate) treatment notably increased the plant height of *W. trilobata* under low-light conditions, an effect which was not observed in *W. chinensis* (Figure 1C).

**Figure 2:**
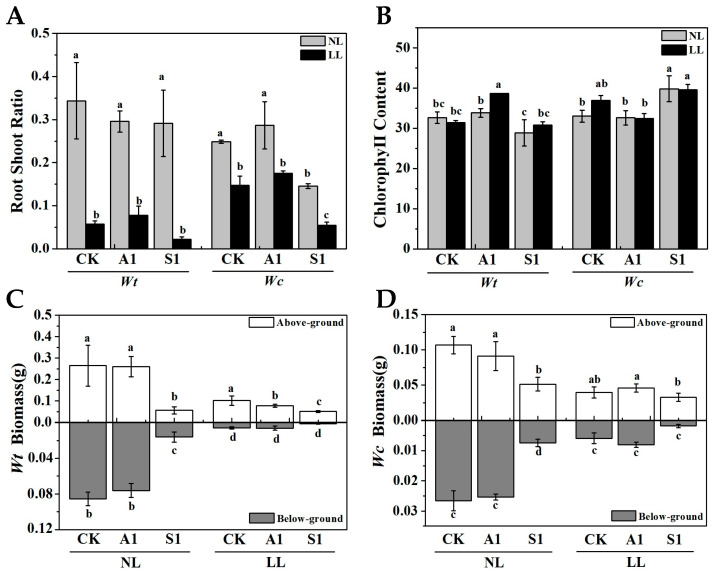
Effects of exogenous ABA and sodium tungstate dihydrate on the root shoot ratio (**A**) chlorophyll content, (**B**) biomass of *W. trilobata*, (**C**) biomass of *W. chinensis*, (**D**) under different light intensities. NL: Normal light, LL: Low light; CK: distilled water with the same amount of ethanol as the A1, A1: 15 μM ABA, S1: 100 μM sodium tungstate dihydrate. The data are presented as mean ± SE (*n* = 3). Different lowercase letters indicate significant differences among treatments according to Duncan’s multiple range test (*p* < 0.05).

The distribution patterns and changes in both aboveground and belowground biomass are crucial indicators of plant adaptation to the environment [28]. For invasive species, shifting biomass allocation to roots or leaves is a strategic response to optimize resource acquisition under fluctuating conditions [29]. The leaf chlorophyll content of *W. trilobata* and *W. chinensis* increased notably after exposure to low-light levels, while both species exhibited significantly reduced aboveground and belowground biomass under low-light conditions compared to normal light (Figure 2). Furthermore, treatment with sodium tungstate resulted in a significant decrease in the biomass of both plants under both normal and low-light conditions, leading to 27.30% and 65.58% decreases in the root-to-shoot ratio under low-light conditions compared to the control group (Figure 2A).

Correlation analysis revealed that under various light intensities, exogenous ABA treatment significantly influenced the growth parameters of both species. In Invasive *W. trilobata*, root length showed a significant positive correlation with leaf number, lateral root number, and leaf area. Similarly, leaf area was significantly and positively correlated with leaf number, root length, and lateral root number. Furthermore, the root-to-shoot ratio exhibited significant positive correlations with leaf number, root length, lateral root number, and leaf area (Table 1, *p* < 0.01). Under various light intensities, sodium tungstate treatment resulted in significant positive correlations between leaf number and plant height, lateral root number, and chlorophyll content in the invasive *W. trilobata*. Additionally, plant height was significantly and positively correlated with the number of lateral roots. Conversely, the root-to-shoot ratio exhibited significant negative correlations with leaf number, chlorophyll content, and specific leaf area (SLA) (Table 2, *p* < 0.01).

For the native *W. chinensis*, exogenous ABA treatment significantly influenced the growth parameters of *W. chinensis* under various light intensities, specific leaf area (SLA) was significantly and positively correlated with chlorophyll content (Table 3, *p* < 0.01). Under sodium tungstate treatment, leaf number and root-to-shoot ratio of *W. chinensis* were significantly and positively correlated with root length, lateral root number, and leaf area when exposed to various light intensities. However, chlorophyll content showed significant negative correlations with leaf number, plant height, root length, lateral root number, leaf area, and root-to-shoot ratio (Table 4, *p* < 0.01).

### 3.2. The Effects of Abscisic Acid on the Photosynthesis of Two Plant Species Under Different Light Intensities

**Figure 3:**
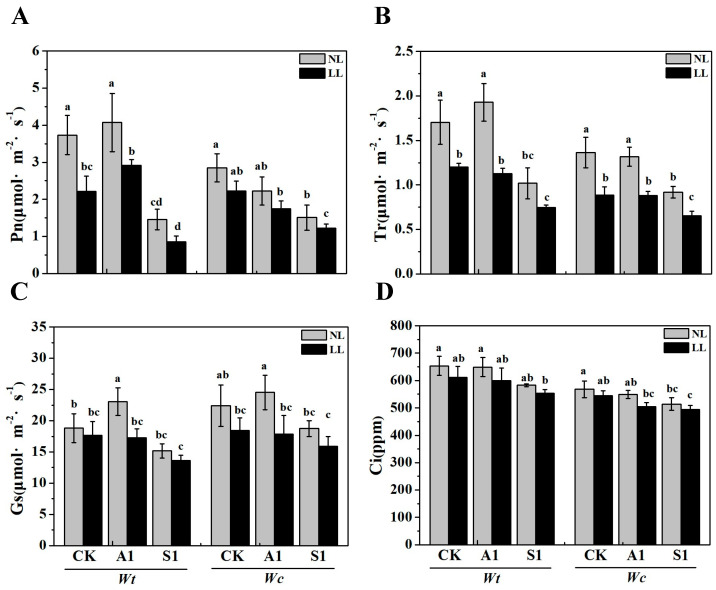
Effects of exogenous ABA and sodium tungstate dihydrate on photosynthesis of *W. trilobata* 2. Concentration. (**A**): photosynthetic rate; (**B**): Transpiration rate; (**C**): Stomatal Conductance; (**D**): intercellular CO_2_ concentration. NL: Normal light, LL: Low light; CK: distilled water with the same amount of ethanol as the A1, A1: 15 μM ABA, S1: 100 μM sodium tungstate dihydrate. The data are presented as mean ± SE (*n* = 3). Different lowercase letters indicate significant differences among treatments according to Duncan’s multiple range test (*p* < 0.05).

As shown in Figure 3, exposing *W. trilobata* and *W. chinensis* to low-light intensity led to a notable decrease in photosynthesis compared to normal light conditions. Specifically, the net photosynthetic rate (*P_n_*), transpiration rate (*T_r_*), stomatal conductance (*g_s_*), and chlorophyll index (*C_i_)* of *W. trilobata* decreased by 40.68%, 29.42%, 6.23%, and 6.37%, respectively. Meanwhile, those parameters for *W. chinensis* decreased by 21.77%, 35.12%, 17.71%, and 2.07%, respectively. The findings indicated that the application of ABA under low-light conditions resulted in a significant increase in the *P_n_*) of *W. trilobata* compared to the low-light control group (Figure 3A), whereas the *P_n_* and *C_i_* of *W. chinensis* exhibited significant decreases (Figure 3A,D). Furthermore, when subjected to low-light intensity, sodium tungstate treatment notably suppressed the photosynthetic activity of both plant species. Specifically, compared to the control group, the *P_n_* of *W. trilobata* decreased by 77.16%, while that of *W. chinensis* was reduced by 57.12% (Figure 3A).

Correlation analysis indicated that the *P_n_* of *W. trilobata* exhibited a positive correlation with the number of lateral roots and the root-to-shoot ratio across varying light intensities and ABA treatments. Moreover, the *T_r_* showed a positive correlation with leaf number, root length, lateral root number, leaf area, and root-to-shoot ratio (Table 1, *p* < 0.01). Similarly, the *T_r_* of *W. chinensis* was positively associated with root length and root-to-shoot ratio, while the intercellular CO_2_ concentration (*C_i_*) correlated positively with leaf area (Table 3, *p* < 0.01).

In the context of diverse light intensities and sodium tungstate treatments, significant positive correlations were observed between the *P_n_* and *C_i_* of *W. trilobata* and the number of leaves and lateral roots. Furthermore, the *T_r_* demonstrated positive correlations with leaf number, lateral root number, and chlorophyll content, while *g_s_* positively correlated with lateral root number (Table 2, *p* < 0.01). Concerning *W. chinensis*, the *P_n_* showed positive correlations with leaf number, root length, lateral root number, leaf area, and root-to-shoot ratio. Additionally, the *T_r_* was positively correlated with root length and root-to-shoot ratio, while the *C_i_* exhibited positive correlations with leaf number, lateral root number, leaf area, and root-to-shoot ratio (Table 4, *p* < 0.01).

### 3.3. The Effects of Abscisic Acid on the Antioxidant Systems of Two Plant Species Under Different Light Intensities

**Figure 4:**
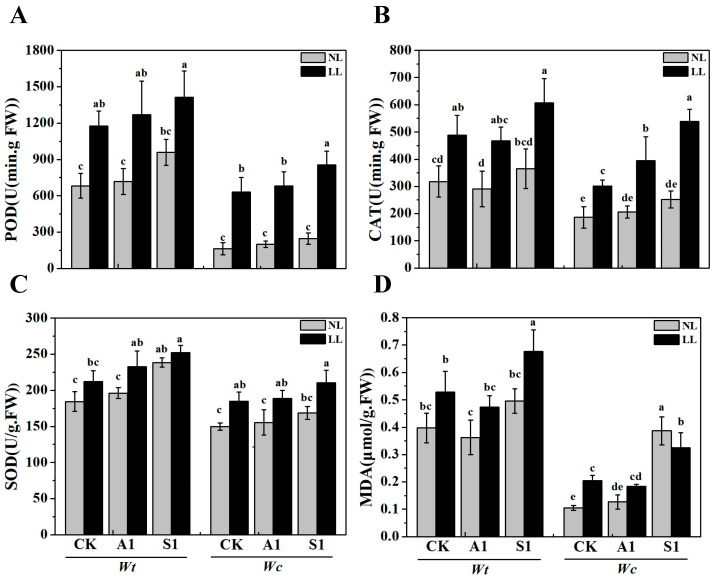
Effects of applying exogenous ABA and sodium tungstate dihydrate on the antioxidant system of *W. trilobata* and *W. chinensis* under different light intensities. (**A**) Peroxidase; (**B**) catalase; (**C**) superoxide dismutase; and (**D**) malondialdehyde. NL: Normal light, LL: Low light; CK: distilled water with the same amount of ethanol as the A1, A1: 15 μM ABA, S1: 100 μM sodium tungstate dihydrate. The data are presented as mean ± SE (*n* = 3). Different lowercase letters indicate significant differences among treatments according to Duncan’s multiple range test (*p* < 0.05).

As a key stress hormone, exogenous ABA can enhance the plant’s antioxidant defense system by upregulating the activities of key enzymes such as superoxide dismutase (SOD), peroxidase (POD), and catalase (CAT). This hormonal regulation is highly light-dependent [11]. As illustrated in Figure 4, the antioxidant enzyme activities and malondialdehyde (MDA) content in the leaves of *W. trilobata* and *W. chinensis* significantly increased following exposure to low-light conditions compared to normal light conditions. When compared to the control group under low-light intensity, the levels of peroxidase (POD), catalase (CAT), and superoxide dismutase (SOD) in *W. trilobata* leaves exhibited a notable increase, while the MDA content markedly decreased with the application of exogenous ABA (Figure 4, *p* < 0.05). In addition, the levels of POD, CAT, SOD, and MDA in *W. chinensis* leaves treated with S1 showed a significant increase under low-light intensity. While the CAT levels in *W. chinensis* leaves treated with ABA were notably elevated, the changes in POD and SOD contents were not significant (Figure 4A–C). The observed differences in enzyme activity between the two plant species following low-light and ABA treatments suggest that ABA application may offer greater benefits to *W. trilobata* in mitigating oxidative damage induced by low-light conditions.

Correlation analysis indicated that the application of ABA under different light intensities was significantly associated with negative correlations between the activities of CAT and POD in *W. trilobata* and various plant characteristics, such as leaf number, lateral root number, leaf area, and root-to-shoot ratio. Additionally, the MDA content showed a negative correlation with root length, lateral root number, and leaf area (Table 1, *p* < 0.01). Furthermore, the leaf SOD activity exhibited negative correlations with leaf number and leaf area. For *W. chinensis*, the levels of POD, SOD, and MDA were negatively correlated with root length, leaf area, and root-to-shoot ratio. Notably, a significant negative correlation was observed between root CAT activity and root length (Table 3, *p* < 0.01).

Under various light intensities, sodium tungstate treatment led to significant negative correlations between peroxidase (POD) activity and both leaf number and chlorophyll content in the invasive *W. trilobata* (Table 2, *p* < 0.01). Similarly, catalase (CAT) activity was significantly and negatively correlated with leaf number, while superoxide dismutase (SOD) activity showed significant negative correlations with leaf number, plant height, lateral root number, and chlorophyll content. Furthermore, malondialdehyde (MDA) content exhibited significant negative correlations with leaf number, lateral root number, and chlorophyll content. In contrast, both CAT activity and MDA content were significantly and positively correlated with the root-to-shoot ratio (Table 2, *p* < 0.01). In the native *W. chinensis*, both CAT and POD activities were significantly and negatively correlated with the root-to-shoot ratio (Table 4, *p* < 0.01). Additionally, SOD activity was significantly and negatively correlated with root length and root-to-shoot ratio. MDA content in the native species showed significant negative correlations with leaf number, root length, plant height, lateral root number, and leaf area, while exhibiting a significant positive correlation with chlorophyll content (Table 4, *p* < 0.01). Results from the two-way ANOVA indicated that the interaction between exogenous ABA and light intensity significantly affected the leaf area, chlorophyll content, and the number of lateral roots in *W. trilobata* (Table 5, *p* < 0.05). Meanwhile, the interaction between sodium tungstate and light intensity significantly influenced the root length, POD activity, and CAT activity in *W. chinensis* (Table 6, *p* < 0.05). These findings imply that the physiological responses of both species to light intensity are highly dependent on the modulation of the ABA signaling pathway.

## 4. Discussion

### 4.1. Morphological Plasticity and Biomass Allocation Under Light Stress

Plant morphology and biomass allocation serve as visible and crucial indicators of a plant’s response to environmental fluctuations [30]. Changes in environmental conditions, particularly light intensity, exert a profound impact on plant development. For instance, severe shading significantly inhibits the growth of various plant seedlings, leading to reduced leaf biomass, root-to-shoot ratios, and total biomass. Such morphological adjustments reflect a trade-off between light capture and resource conservation, which is especially pronounced in invasive species competing for space in heterogeneous forest understories [28,31]. Consistent with these findings, our study demonstrates that low-light conditions significantly inhibited the growth of both the invasive *W. trilobata* and the native *W. chinensis*. This inhibition was evident in reduced leaf characteristics (leaf number, leaf area, and specific leaf area) (Figure 1A,B,D), restricted root development (root length and lateral root number) (Figure 1E,F), and a substantial decrease in total biomass (Figure 2). In this study, sodium tungstate was utilized as a chemical inhibitor of ABA biosynthesis. While sodium tungstate effectively suppresses ABA accumulation by targeting molybdenum-containing aldehyde oxidases, its absolute specificity is a known limitation. High doses of tungsten may interfere with other molybdenum-dependent enzymes, such as nitrate reductase, or even trigger heavy metal stress responses [32]. To minimize these confounding variables, we employed a low concentration (100 μM), which was sufficient to effectively suppress ABA biosynthesis while simultaneously avoiding non-specific side effects or growth retardation. Consistent with this targeted inhibition, the addition of sodium tungstate intensified the inhibitory effects of low-light stress [21,33]. This treatment led to a severe reduction in both aboveground and belowground biomass in *W. trilobata*, as well as a significant decrease in the belowground biomass of *W. chinensis* (Figure 2C,D). Interestingly, the S1 treatment specifically increased the plant height of *W. trilobata* under low-light conditions (Figure 1C), highlighting the intricate interplay between environmental light factors and internal hormonal responses in shaping plant developmental plasticity [16,34].

### 4.2. Photosynthetic Coordination and ABA-Mediated Regulation

Under limited radiation, plants often increase their chlorophyll content to enhance light absorption and promote growth maintenance [35]. In our study, the chlorophyll content of the native *W. chinensis* significantly increased under low light conditions compared to normal conditions, whereas the invasive *W. trilobata* showed no significant change unless treated with exogenous ABA (Figure 2B). Generally, low-light intensity diminishes stomatal conductance (*g_s_*), transpiration rate (*T_r_*), and photosynthetic enzyme activity [36]. Accordingly, we observed that the net photosynthetic rate (*P_n_*), *g_s_*, *T_r_*, and intercellular CO_2_ concentration (*C_i_*) of both species were lower under low light than under normal light (Figure 3). However, species-specific responses emerged upon hormonal manipulation. Exogenous ABA treatment significantly increased the *P_n_* of the invasive *W. trilobata* under low light, while high concentrations of ABA effectively reduced its *g_s_* and *T_r_*, indicating superior stomatal regulation [27,36]. In contrast, inhibiting endogenous ABA with sodium tungstate resulted in severe suppression of photosynthesis in both species. These findings suggest that *W. trilobata* relies heavily on ABA signaling to regulate stomatal dynamics and maintain photosynthetic efficiency under adverse conditions [22].

### 4.3. Antioxidant Defense and Oxidative Stress Mitigation

Under environmental stress, the balance between reactive oxygen species (ROS) and the antioxidant system is disrupted, leading to lipid peroxidation, increased malondialdehyde (MDA) content, and a subsequent upregulation of antioxidant enzymes such as POD, CAT, and SOD [37,38]. In the present study, low-light stress induced oxidative damage, significantly increasing the MDA content and the activities of POD, CAT, and SOD in the leaves of both *W. trilobata* and *W. chinensis* (Figure 4). The application of exogenous abscisic acid (ABA) effectively alleviated these detrimental effects. Consistent with the pivotal role of the ABA machinery in stress tolerance [11], our results demonstrated that ABA application in *W. trilobata* mitigated oxidative damage by reducing the excessive increase in CAT activity and MDA content. Conversely, sodium tungstate treatment exacerbated the oxidative stress, further elevating antioxidant enzyme activities and MDA accumulation. This enhanced antioxidant defense mechanism, modulated by ABA, likely prevents oxidative damage to chloroplasts, thereby maintaining higher *P_n_* under low-light conditions—a protective mechanism similarly observed in other plant species under severe environmental stress [10,27].

### 4.4. Ecological Implications for Invasion Success and Management

ABA is a core phytohormone that responds to adversities such as drought, salinity, and low light by coordinating stomatal closure, photosynthesis, and antioxidant systems [10,39]. Exogenous ABA application and the use of synthesis inhibitors (like sodium tungstate) provide a crucial window into the physiological adaptation mechanisms of invasive plants. Our comparative experiments reveal a significantly more pronounced protective effect of ABA on the invasive species *W. trilobata* compared to its native congener. This differential sensitivity indicates that the invasive plant heavily relies on specific endogenous hormone levels and rapid ABA signaling pathways to adapt to fluctuating environments [25,26]. While traditional ecological views often characterize highly invasive plants as strictly light-demanding, emerging evidence indicates that successful invaders can dynamically adjust their ABA profiles to acclimate to severe shading [33]. The superior photosynthetic recovery of *W. trilobata* under ABA treatment suggests a sophisticated fine-tuning mechanism rather than a simple stress response. While ABA typically triggers OST1-mediated stomatal closure via SLAC1/SLAH3 channels [17], *W. trilobata* maintained an optimal balance between CO_2_ supply and light-use efficiency. This indicates possibly a high level of physiological plasticity in its ABA signaling network, allowing for the induction of photoprotective proteins and thylakoid stabilization without excessive stomatal limitation. In contrast, *W. chinensis* exhibited ABA-suppressed *P_n_*, likely due to uncoordinated stomatal sensitivity or insufficient antioxidant capacity in the mesophyll. Similar ABA-mediated plasticity has been documented in other successful invaders like *Mikania micrantha*, where rapid hormonal modulation facilitates adaptation to fluctuating light niches [23]. Collectively, the heightened sensitivity of *W. trilobata* to ABA highlights its evolutionary advantage in reconfiguring photosynthetic machinery for optimal resource allocation as well as enhanced antioxidant defense under light-limiting conditions, further contributing to its invasive success. Recognizing this strong ABA dependence also provides a theoretical basis for ecological restoration. For instance, strategies that interfere with ABA synthesis or signal transduction in *W. trilobata* could effectively weaken its competitiveness and limit its spread under environmental changes.

## 5. Conclusions

In conclusion, this study demonstrates that the phytohormone abscisic acid (ABA) serves as a critical physiological integrator that mediates the adaptation of the invasive plant *W. trilobata* to low-light environments. Our comparative analysis reveals that while low light suppresses the overall growth and photosynthetic capacity of both species, *W. trilobata* exhibits superior physiological plasticity through its ABA-mediated regulatory network. Specifically, exogenous ABA application significantly alleviates the reduction in net photosynthetic rate (*P_n_*) and biomass in *W. trilobata* by optimizing stomatal dynamics and enhancing its antioxidant defense system (SOD, POD, and CAT), thereby effectively mitigating lipid peroxidation (MDA). Conversely, the severe growth inhibition and exacerbated oxidative damage observed when ABA biosynthesis was blocked by sodium tungstate underscore the vital dependence of *W. trilobata* on endogenous ABA signaling for maintaining its competitive advantage. These findings indicate that the invasive success of *W. trilobata* in heterogeneous light habitats is underpinned by its sophisticated and highly responsive ABA-mediated defense mechanisms, providing a potential theoretical basis for ecological management strategies aimed at weakening its environmental resilience.

## Figures and Tables

**Table 1:** Correlation analysis of effects of exogenous ABA on the growth index, photosynthesis and antioxidant system of *W. trilobata* under different light intensity conditions. (LN: number of leaves; PH: plant height; RL: root length; LRN: lateral root number; CC: chlorophyll content; LA: leaf area; SLA: specific leaf area; RSR: root shoot ratio; POD: peroxidase; CAT: catalase; SOD: superoxide dismutase; MDA: malonaldehyde; *P_n_*: photosynthetic rate; *T_r_*: transpiration rate; *g_s_*: stomatal conductance; *C_i_*: intercellular CO_2_ concentration).

	LN	PH	RL	LRN	CC	LA	SLA	RSR	POD	CAT	SOD	MDA	*P_n_*	*T_r_*	*g_s_*	*C_i_*
LN	1	−0.023	0.776 **	0.611 *	−0.471	0.753 **	−0.378	0.807 **	−0.816 **	−0.718 **	−0.809 **	−0.536	0.485	0.795 **	0.227	0.439
PH		1	−0.412	−0.586 *	−0.04	−0.38	0.373	−0.422	0.041	0.219	0.036	0.35	−0.487	−0.44	−0.445	−0.278
RL			1	0.792 **	−0.234	0.828 **	−0.670 *	0.853 **	−0.699 *	−0.661 *	−0.519	−0.719 **	0.638 *	0.906 **	0.556	0.377
LRN				1	−0.107	0.882 **	−0.644 *	0.829 **	−0.720 **	−0.786 **	−0.554	−0.818 **	0.806 **	0.815 **	0.658 *	0.659 *
CC					1	−0.475	−0.208	−0.334	0.371	0.203	0.532	−0.063	0.06	−0.291	−0.059	−0.17
LA						1	−0.529	0.807 **	−0.835 **	−0.790 **	−0.746 **	−0.750 **	0.673 *	0.872 **	0.681 *	0.636 *
SLA							1	−0.583 *	0.445	0.499	0.347	0.665 *	−0.57	−0.526	−0.352	−0.473
RSR								1	−0.808 **	−0.831 **	−0.618 *	−0.604 *	0.800 **	0.763 **	0.462	0.46
POD									1	0.900 **	0.809 **	0.664 *	−0.705 *	−0.729 **	−0.593 *	−0.43
CAT										1	0.681 *	0.605 *	−0.861 **	−0.649 *	−0.593 *	−0.503
SOD											1	0.612 *	−0.385	−0.630 *	−0.246	−0.449
MDA												1	−0.579 *	−0.712 **	−0.554	−0.385
*P_n_*													1	0.596 *	0.700 *	0.524
*T_r_*														1	0.629 *	0.523
*g_s_*															1	0.32
*C_i_*																1

The symbolic of “*” indicates significant correlation (*p* < 0.05), and “**” indicates extremely significant correlation (*p* < 0.01).

**Table 2:** Correlation analysis of effects of sodium tungstate dihydrate on the growth index, photosynthesis and antioxidant system of *W. trilobata* under different light intensity conditions. (The symbols are explained in Table 1).

	LN	PH	RL	LRN	CC	LA	SLA	RSR	POD	CAT	SOD	MDA	*P_n_*	*T_r_*	*g_s_*	*C_i_*
LN	1	0.733 **	0.326	0.934 **	0.785 **	−0.583 *	0.612 *	−0.723 **	−0.808 **	−0.724 **	−0.939 **	−0.788 **	0.923 **	0.907 **	0.703 *	0.890 **
PH		1	0.4	0.766 **	0.507	−0.274	0.487	−0.414	−0.319	−0.195	−0.744 **	−0.361	0.696 *	0.638 *	0.690 *	0.689 *
RL			1	0.459	−0.229	0.506	−0.458	0.334	−0.193	0.079	−0.406	−0.1	0.43	0.389	0.264	0.244
LRN				1	0.671 *	−0.423	0.51	−0.587 *	−0.665 *	−0.551	−0.924 **	−0.744 **	0.930 **	0.936 **	0.762 **	0.844 **
CC					1	−0.941 **	0.931 **	−0.960 **	−0.712 **	−0.672 *	−0.713 **	−0.760 **	0.677 *	0.714 **	0.693 *	0.695 *
LA						1	−0.945 **	0.959 **	0.608 *	0.686 *	0.476	0.678 *	−0.467	−0.496	−0.503	−0.5
SLA							1	−0.927 **	−0.485	−0.555	−0.529	−0.580 *	0.521	0.497	0.588 *	0.533
RSR								1	0.635 *	0.723 **	0.610 *	0.773 **	−0.615 *	−0.652 *	−0.634 *	−0.599 *
POD									1	0.844 **	0.688 *	0.715 **	−0.787 **	−0.737 **	−0.506	−0.726 **
CAT										1	0.526	0.791 **	−0.660 *	−0.618 *	−0.358	−0.600 *
SOD											1	0.704 *	−0.840 **	−0.864 **	−0.604 *	−0.870 **
MDA												1	−0.674 *	−0.848 **	−0.612 *	−0.649 *
*P_n_*													1	0.870 **	0.775 **	0.791 **
*T_r_*														1	0.787 **	0.863 **
*g_s_*															1	0.591 *
*C_i_*																1

The symbolic of “*” indicates significant correlation (*p* < 0.05), and “**” indicates extremely significant correlation (*p* < 0.01).

**Table 3:** Correlation analysis of effects of exogenous ABA on the growth index, photosynthesis and antioxidant system of *W. chinensis* under different light intensity conditions. (The symbols are explained in Table 1).

	LN	PH	RL	LRN	CC	LA	SLA	RSR	POD	CAT	SOD	MDA	*P_n_*	*T_r_*	*g_s_*	*C_i_*
LN	1	0.219	0.13	0.252	−0.438	0.441	−0.478	0.296	−0.514	−0.209	−0.151	−0.348	0.391	0.188	0.096	0.383
PH		1	0.441	−0.09	−0.532	0.22	−0.46	0.336	−0.254	−0.225	−0.402	−0.525	0.006	0.168	−0.169	0.011
RL			1	0.485	−0.256	0.598 *	−0.517	0.683 *	−0.807 **	−0.804 **	−0.822 **	−0.859 **	0.604 *	0.871 **	0.674 *	0.498
LRN				1	−0.331	0.133	−0.295	0.554	−0.618 *	−0.627 *	−0.444	−0.486	0.3	0.645 *	0.489	0.157
CC					1	−0.15	0.746 **	−0.487	0.408	0.255	0.359	0.525	0.278	−0.339	−0.208	0.051
LA						1	−0.435	0.701 *	−0.748 **	−0.591 *	−0.768 **	−0.720 **	0.664 *	0.643 *	0.532	0.787 **
SLA							1	−0.661 *	0.527	0.289	0.444	0.667 *	−0.138	−0.495	−0.423	−0.021
RSR								1	−0.796 **	−0.633 *	−0.848 **	−0.885 **	0.322	0.767 **	0.576	0.404
POD									1	0.816 **	0.819 **	0.839 **	−0.561	−0.880 **	−0.759 **	−0.719 **
CAT										1	0.841 **	0.741 **	−0.518	−0.761 **	−0.618 *	−0.702 *
SOD											1	0.902 **	−0.441	−0.855 **	−0.645 *	−0.615 *
MDA												1	−0.487	−0.841 **	−0.564	−0.504
*P_n_*													1	0.535	0.357	0.617 *
*T_r_*														1	0.856 **	0.509
*g_s_*															1	0.497
*C_i_*																1

The symbolic of “*” indicates significant correlation (*p* < 0.05), and “**” indicates extremely significant correlation (*p* < 0.01).

**Table 4:** Correlation analysis of effects of sodium tungstate dihydrate on the growth index, photosynthesis and antioxidant system of *W. chinensis* under different light intensity conditions. (The symbols are explained in Table 1).

	LN	PH	RL	LRN	CC	LA	SLA	RSR	POD	CAT	SOD	MDA	*P_n_*	*T_r_*	*g_s_*	*C_i_*
LN	1	0.687 *	0.718 **	0.847 **	−0.618 *	0.746 **	0.167	0.627 *	−0.295	−0.43	−0.304	−0.764 **	0.872 **	0.545	0.448	0.709 **
PH		1	0.861 **	0.719 **	−0.767 **	0.675 *	−0.039	0.690 *	−0.309	−0.468	−0.468	−0.808 **	0.703 *	0.628 *	0.39	0.447
RL			1	0.850 **	−0.770 **	0.785 **	−0.054	0.885 **	−0.501	−0.676 *	−0.719 **	−0.879 **	0.884 **	0.871 **	0.655 *	0.707 *
LRN				1	−0.762 **	0.622 *	0.331	0.756 **	−0.365	−0.620 *	−0.516	−0.863 **	0.857 **	0.703 *	0.534	0.720 **
CC					1	−0.709 **	−0.114	−0.723 **	0.456	0.496	0.545	0.886 **	−0.649 *	−0.689 *	−0.589 *	−0.618 *
LA						1	−0.104	0.723 **	−0.425	−0.461	−0.549	−0.801 **	0.852 **	0.671 *	0.638 *	0.798 **
SLA							1	−0.214	0.517	0.174	0.349	−0.212	0.015	−0.206	−0.034	0.027
RSR								1	−0.805 **	−0.888 **	−0.880 **	−0.721 **	0.866 **	0.911 **	0.752 **	0.737 **
POD									1	0.856 **	0.847 **	0.272	−0.508	−0.756 **	−0.649 *	−0.577 *
CAT										1	0.905 **	0.412	−0.690 *	−0.799 **	−0.631 *	−0.667 *
SOD											1	0.459	−0.641 *	−0.848 **	−0.656 *	−0.608 *
MDA												1	−0.818 **	−0.660 *	−0.55	−0.659 *
*P_n_*													1	0.744 **	0.609 *	0.823 **
*T_r_*														1	0.880 **	0.707 *
*g_s_*															1	0.647 *
*C_i_*																1

The symbolic of “*” indicates significant correlation (*p* < 0.05), and “**” indicates extremely significant correlation (*p* < 0.01).

**Table 5:** Variance analysis results of the effects of exogenous ABA and sodium tungstate on *W. trilobata* under different light intensities according to Duncan’s multiple range test.

*Wt* Parameters	ABA × Light Intensity		Sodium Tungstate × Light Intensity	
	*F*	*p*	*F*	*p*
LN	7.556	0.01	57	<0.001
PH	1.138	0.39	32.013	<0.001
RL	11.436	0.003	19.739	<0.001
LRN	22.17	<0.001	86.83	<0.001
CC	35.313	<0.001	120.664	<0.001
LA	45.829	<0.001	709.164	<0.001
SLA	5.311	0.026	70.253	<0.001
RSR	28.808	<0.001	91.473	<0.001
POD	9.759	0.005	13.738	0.002
CAT	8.069	0.008	9.291	0.006
SOD	5.413	0.025	19.621	<0.001
MDA	4.76	0.035	9.533	0.005
*P_n_*	7.706	0.01	33.708	<0.001
*T_r_*	16.332	0.001	20.651	<0.001
*g_s_*	4.9	0.032	5.371	0.026
*C_i_*	1.446	0.3	7.465	0.01

Notes: *p* < 0.05 indicates a significant difference, *p* > 0.05 means no significant difference.

**Table 6:** Variance analysis results of the effects of exogenous ABA and sodium tungstate on *W. chinensis* under different light intensities according to Duncan’s multiple range test.

*Wc* Parameters	ABA × Light Intensity		Sodium Tungstate × Light Intensity	
	*F*	*p*	*F*	*p*
LN	1.333	0.33	7.583	0.01
PH	1.648	0.254	6.025	0.019
RL	9.908	0.005	30.471	<0.001
LRN	3.138	0.087	35.119	<0.001
CC	6.363	0.016	7.5	0.01
LA	3.579	0.066	7.627	0.01
SLA	19.581	<0.001	10.581	0.004
RSR	14.208	0.001	130.374	<0.001
POD	29.623	<0.001	40.619	<0.001
CAT	10.641	0.004	55.995	<0.001
SOD	7.698	0.01	14.424	0.001
MDA	22.242	<0.001	31.127	<0.001
*P_n_*	6.05	0.019	18.79	0.001
*T_r_*	16.178	0.001	23.421	<0.001
*g_s_*	3.888	0.055	4.494	0.04
*C_i_*	5.179	0.028	6.581	0.015

Notes: *p* < 0.05 indicates a significant difference, *p* > 0.05 means no significant difference.

## Data Availability

The original contributions presented in this study are included in the article. Further inquiries can be directed to the corresponding author.
